# Clinical effects of different center of rotation reconstructions in total hip arthroplasty after femoral neck fractures: a cohort study including a follow-up analysis on patient’s mobility and daily living ability

**DOI:** 10.1186/s10195-023-00738-y

**Published:** 2023-11-09

**Authors:** Christopher Nieschk, Johanna Abelmann-Brockmann, Leonard Lisitano, Annabel Fenwick, Heinz Röttinger, Michael Ecker, Edgar Mayr, Timon Röttinger

**Affiliations:** 1 Universitätsklinikum Augsburg, Klinik für Unfallchirurgie, Orthopädie, Plastische und Handchirurgie, Stenglinstraße 2, 86156 Augsburg, Deutschland; 2 München Klinik Neuperlach, Oskar-Maria-Graf-Ring 51, 81737 München, Deutschland; 3 Faculty of Medical University of Pleven, Pleven, Bulgaria

**Keywords:** Center of rotation, Total hip arthroplasty, Femoral neck fracture, Parker mobility score, Cup position

## Abstract

**Background:**

The aim of this study is a clinical evaluation of the center of rotation (COR) placement towards a patient’s recovery with respect to daily living ability and mobility. In past experiments based on three-dimensional (3D) models, medialization of the COR in total hip arthroplasty (THA) showed a negative influence on muscle strength of the abductors and reaction force of the hip joint. This contradicts paradigms, where reduced hip loading forces are claimed to increase functional outcomes.

**Methods:**

The plain X-rays of 110 patients who underwent THA after a femoral neck fracture between January 2019 and January 2021 were retrospectively evaluated. A Barthel Index on discharge was obtained in 69 cases. 47 patients were available for a follow-up interview concerning the Barthel Index, Parker mobility score (PMS), and pain levels (NRS) 6 and 12 months after surgery.

**Results:**

Medialization of the COR had a significantly negative effect on the need for care (Barthel Index) at patient discharge (Spearman correlation 0.357, *p* = 0.013). The effect on the PMS is still existent at 6 and 12 months (Spearman correlation 0.471, *p* = 0.009 at 6 months; 0.472, *p* = 0.008 at 12 months). Mann–Whitney *U* tests showed that the groups with medialized COR performed significantly worse than the lateralized groups. This was seen for the Barthel Index at discharge and at 6 months after surgery and for the PMS at 6 and 12 months. The accurately reconstructed CORs showed no significant differences from the lateralized rotation centers in need of care and mobility.

The superior COR placement group showed significantly reduced mobility at 12 months in contrast to the inferior COR placement group (*p* = 0.008), and the group of accurately reconstructed rotation centers showed significantly less pain than the inferior COR placement group (*p* = 0.007 after 6 months, *p* = 0.026 after 12 months). Especially the combination of both (superomedialization) leads to reduced mobility (Spearman correlation 0.67, *p* =  < 0.001).

**Conclusions:**

COR superior displacement, COR medialization, and the combination of both (superomedialization, Spearman *p* =  < 0.001) lead to reduced mobility while inferior displacement showed increased pain. According to our results, we recommend an exact vertical COR restoration, while horizontal medial displacement needs to be avoided.

***Level of evidence*:**

III.

## Introduction

The medial femoral neck fracture is considered an indicator fracture for osteoporosis and predominantly affects the aging patient. According to our in-house standards, total hip arthroplasty (THA) is offered to active patients with high functional demands. This needs thorough evaluation, as the alternative hemiarthroplastic treatment (HHA) is mainly used for geriatric patients with low functional requirements and high intraoperative risk. In this case, attention to comorbidities and a shorter operating time justify the less invasive nature of the procedure, although THA achieves better functional results and is associated with a higher quality of life [1]. Despite increased early complications compared with HHA, patients with THA also show a lower susceptibility to pain in addition to better postoperative mobility [2]. Comorbid patients also show significantly higher dislocation rates after THA [3].

The recovery of the daily living ability and optimal management of potential comorbidities is the central aim in the treatment of osteoporotic indicator fractures in the aging patient. Overall functional rehabilitation represents an important evaluation criterion in geriatric traumatology [4]. Because of socioeconomic considerations, possible prevention of long-term care dependency, and the ethical assignment of a surgeon, it is particularly necessary to analyze our care strategies to regular quality assuring data analysis. This study analyzes the influence of an accurate center of rotation (COR) reconstruction on the recovery of daily living ability. The Barthel Index [5] and the Parker mobility score (PMS) [6] are suitable for estimating mobility and care independence. The PMS is known to have high intertester reliability [7] and it allows a thorough prediction of the in-hospital rehabilitation potential after hip fracture surgery [8]. In clinical practice, the Barthel Index is particularly suitable for assessing the need for post-inpatient care and determines the rehabilitation prognosis, the type of rehabilitation, and, in the long term, the further care and nursing dependency. Even if not all abilities assessed by the Barthel Index are related to the functionality of the hip joint, experience shows that limited mobility and functionality of the lower extremity affects the patient in many ways and in other areas of life. Data shows that limited mobility aggravates comorbidities and increases mortality [9–11]. Advanced age, low preoperative functional status, ability to participate in physical therapy, and blood loss appear to be particularly outcome relevant in the management of hip fractures [12]. Overall, patients with femoral neck fractures seem to be a vulnerable patient group, since they show a significant higher mortality, as well as septic and aseptic failure rate compared with patients receiving THA for treatment of osteoarthritis [13].

In our facility, patients who undergo THA receive full weight-bearing exercise from day 1 postoperatively. For all treatments of aging patients, the rapid return to daily living ability is an essential primary goal. In this study, patients with THA after medial femoral neck fracture are analyzed for a possible influence of anatomical reconstruction of the center of rotation (COR) on regaining daily living skills. The expected patient population consists predominantly of aging patients with functional requirements and manageable comorbidities. In our opinion, an optimal surgical outcome is essential for maintaining the quality of life of these patients. In experiments based on 3D models of dysplastic hips, COR medialization showed a negative influence on the muscle strength of the abductors and the reaction force of the hip joint [14]. The COR displacement in these experiments were referenced to the healthy nondysplastic hip, and these results could therefore also be relevant for other indications of THA, such as femoral neck fractures. It also contradicts claims were inferomedial. COR placement is attributed to be advantageous with respect to functional results [15, 16].

## Methods and materials

### Ethical approval and patient collective

In this retrospective follow-up study, patients at a level I trauma center (University Hospital) who underwent THA for treatment of traumatic femoral neck fractures between January 2019 and January 2021 were evaluated. All patients were surgically treated using a transgluteal Bauer approach in lateral position. Only cases with surgery performed by senior and chief physicians were included in the study. In these cases, residents were only involved in assisting positions.

The data was retrospectively collected using records of the patients and by using telephone follow-up interviews to evaluate the outcome and quality of the surgical treatment. The data was irreversibly anonymized. No experiments on patients were carried out. The physical integrity and the treatment of the patients were not affected by the data collection.

**Fig. 1:**
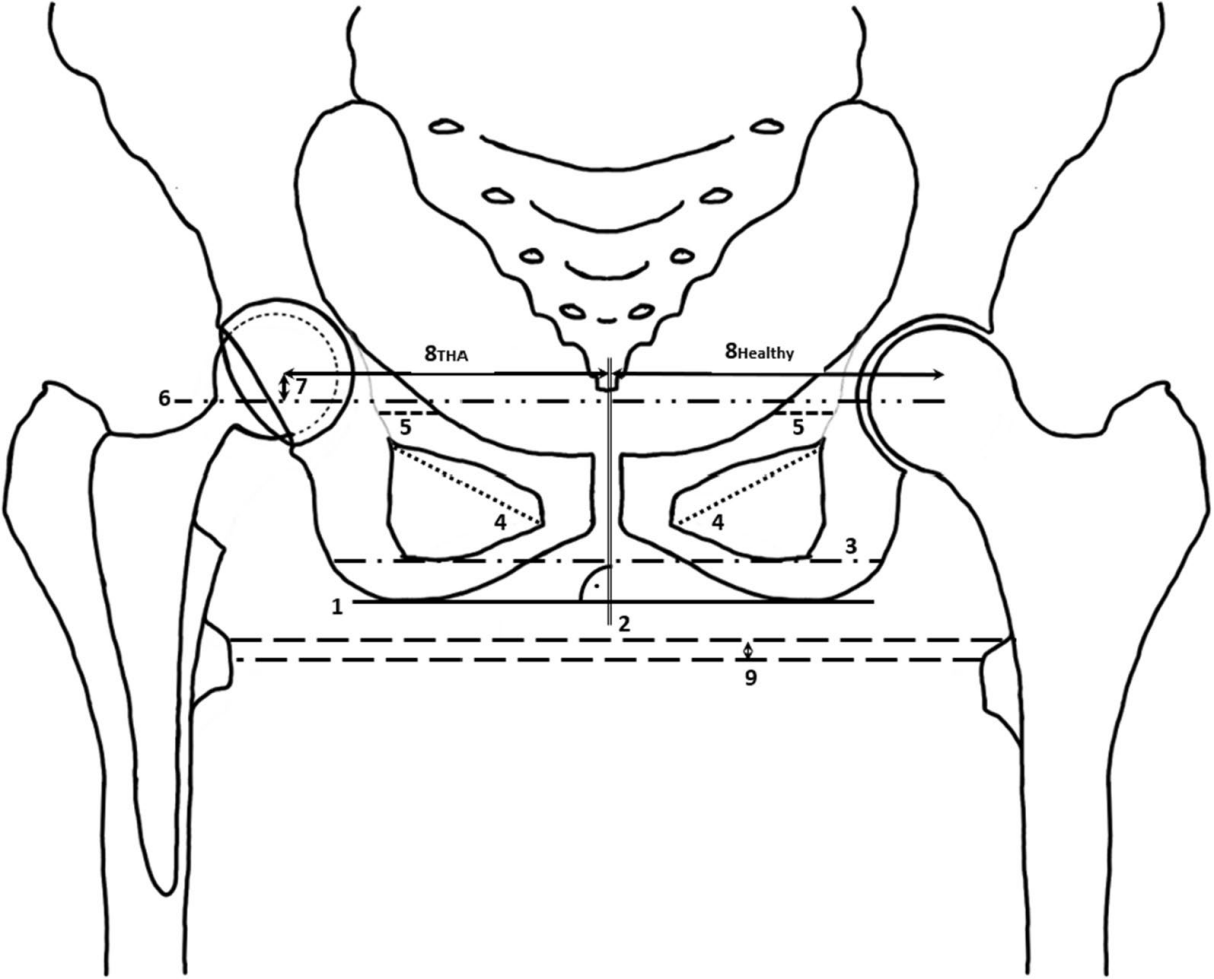
Sketch of a pelvic overview. 1–5: Marks of quality requirements, 6: parallel to 1, 7: RC-SI, 8: THA and healthy horizontal distances, RC-ML = 8THA—8healthy, 9: anatomical leg length difference

All applicable cases were screened for inclusion and exclusion criteria before study enrollment. In the recruitment procedure, pathological fractures and history of surgical procedures of both hips, including conversion osteotomies of the pelvis, femur, and knee were excluded. Patients with conditions that made proximal femoral replacements necessary were excluded, since high complication rates were reported in past studies [17]. In addition, a suitable postoperative radiograph meeting the quality requirements had to be available. The radiograph was checked for projection and rotation errors in the two-dimensional (2D) image. Figure 1 shows that the two deepest points of the ischiadic tuberosity were connected by a straight line (line 1), which is exactly perpendicular to the midline mark of the symphysis (line 2). At the same time, the symphysis marking had to intersect a drawn line from both outer edges of the ischial ramus, which passes over the deepest point of the obturator foramen (line 3) at its midpoint. Finally, both foramina were checked for size equality by diameter (line 4), and the distances from the projected linea iliopectinea and linea ilioischiadica (line 5) were measured. Deviations of the mentioned quality criteria > 1 mm also led to complete case exclusion, so that a study population of 110 patients was examined. A discrepancy to the opposite side > 0.1 mm ensured deliberate exclusion of the horizontal center-of-rotation position (RC-ML) (see below and Fig. 1) from further data analysis. This was done to mitigate the risk of inaccurate results. Due to the retrospective follow-up design of the study, the data set could not be collected completely for each patient, so the number varied (Table 1).

**Table 1:** Overview of all variables collected including case numbers of the different endpoints and standard deviations and of all total endoprostheses used in the patient collective (Zimmer Biomet)

	Total *N*	Incl. RCML	Barthel Index upon discharge	Thereof incl. RCML	Follow-up	Thereof incl. RCML
*N*	110	86	68	53	47	43
Sex (M/W)	M: 29 (26.4%) W: 81 (73.6%)	M: 17 (19.8%) W: 69 (80.2%)	M: 19 (27.9%) W: 49 (72.1%)	M: 10 (18.9%) W: 43 (81.1%)	M: 12 (25.5%) W: 35 (74.5%)	M: 11 (25.6%) W: 32 (74.4%)
Age (years)	77 ± 8.55	77.12 ± 8.57	80.19 ± 7.50	79.85 ± 7.50	76.87 ± 8.47	76.63 ± 8.65
Size (cm)	167.41 ± 9.43	166.41 ± 9.66	167.96 ± 7.77	167.35 ± 7.87	168.13 ± 8.51	167.97 ± 8.76
Weight (kg)	67.91 ± 14.47	67.30 ± 14.53	68.41 ± 14.96	67.33 ± 15.18	67.17 ± 13.46	67.65 ± 13.97
BMI	24.26 ± 4.22	24.42 ± 3.94	24.26 ± 4.34	24.26 ± 4.07	23.79 ± 3.57	24.04 ± 3.60
RC-ML (mm)	−0.08 ± 4,38	−0.08 ± 4.38	−0.06 ± 4.29	−0.06 ± 4.29	−0.29 ± 5.08	−0.29 ± 5.08
RC-SI (mm)	−1.59 ± 4.71	−1.63 ± 4.54	−2.03 ± 4.29	−2.19 ± 4.20	−1.49 ± 5.19	−1.74 ± 4.96
ALL (mm)	1.03 ± 6.75	0.94 ± 6.73	0.91 ± 6.54	0.83 ± 6.72	0.80 ± 7.62	0.61 ± 7.58
Prosthesis	Shaft		Acetabulum			
M.E. Müller straight stem^®^	82 (74.55%)		–			
Spotorno ^®^ stem	21 (19.09%)		–			
Zweymüller stem ^®^	6 (5.45%)		–			
Revitan ^®^ stem	1 (0.91%)		–			
Allofit^®^ cup	–		98 (89.09%)			
Low profile cup	–		11 (10%)			
Avantage	–		1 (0.91%)			

### Radiological measurement

The evaluated radiographs consisted of anterior–posterior pelvic overviews appropriate for the indication with generous exposure of the femur. The first postoperative radiographs were used to collect all variables. The following measurements are illustrated in Fig. 1. To determine the horizontal (RC-ML) and vertical (RC-SI) center-of-rotation position, the previously placed markers could be adopted to maintain quality requirements. In addition, a parallel line (line 6) was drawn to the straight line of both ischiadic tubera (line 1) starting from the healthy center of rotation, so that the RC-SI (line 7) could be determined from the center of rotation of the total endoprosthesis. Comparison of the horizontal distances of both sides was made by determining both distances (8THA and 8healthy) between the center of rotation and the marker running centrally through the symphysis (line 2). By subtracting 8THA—8healthy, the RC-ML could be formed. As described by Durand-Hill et al., the opposite side can be used in a reliable way for the planning THA [18]. Therefore, in our study, changes in COR are determined using the healthy side to evaluate the original COR position. Lastly, anatomic leg length (ALL) was determined by trochanteric lesser trochanter height differences (line 9).

### Clinical data collection

To ensure that possible unknown influencing factors are not missed, additional variables were collected on the basis of surgical protocols, patient history, and the internal digital data processing program ORBIS^®^ (Agfa Health Care). These sources provided information about the inpatient course, the short-term recovery after surgery, and the need for care after discharge. Thus, the Barthel Index and the type of prosthesis used (Table 1) were considered in the statistical analysis. In our clinical routine, the nursing staff is in charge of determining the Barthel Index at admission and discharge. To achieve a long-term quality assessment mobility, independence and pain were evaluated using the Parker mobility score (PMS), the Barthel Index, and the pain level (NRS) at 6 and 12 months after surgery. This data were acquired using respective follow-up interviews. The follow-up interview was respectively conducted by telephone. Previous studies showed that prefracture mobility is an independent outcome predictor [19]. To analyze the isolated influence of rotation center reconstruction as precise as possible, only patients with full PMS (9/9) and full Barthel Index (100/100) before the fracture event were included in further analysis. This way, an interfering effect can be minimized.

### Statistical analysis

Statistical analysis was performed using SPSS^®^ version 28.0 (IBM, SPSS Inc. Armonk, NY). Due to the ordinal scaled dependent variables, a nonparametric test had to be used. Possible outcome-relevant parameters were identified in a first correlation analysis according to Spearman, in which all variables were entered. For this purpose, RC-ML was additionally indexed to account for individual body relations, which were calculated as follows: RC-MLI = RC-ML/distance line 8healthy.

In our department we seem to have a tendency for superomedial cup positions (Table 1). This discovery of the frequently interrelated reconstruction positioning provided for the development of the CPS score. The formation by adding RC-ML and RC-SI is extremely useful especially for analyzing the milling direction and the effect of this superomedial versus inferolateral cup positions. The score takes on positive values for superior and medial cup positions and negative values for inferior and lateral cup positions. With a shallow and flat milling technique an inferolateral COR reconstruction is almost predetermined, especially with increasing cup size. A deep and steeper milling technique in combination with a small cup predisposes to a superomedial COR position. Patients with an RC-SI greater than 10 mm and RC-ML greater than 6.5 mm were excluded. Deviations of the center of rotation of this magnitude were considered to be clinically extreme displacements and should not artificially attenuate or even enhance a possible correlation.

In addition, three groups for center-of-rotation reconstruction were formed: medial (A), lateral (B), exact (C), and superior (A), inferior (B), exact (C). For consistent comparability, group C included all patients with a reconstruction accuracy of < 2 mm. Mann–Whitney *U* tests for further statistical analysis was performed. The nonparametric test (applicable for ordinally scaled dependent variable) makes putative outliers statistically evaluable. This had the advantage of not having to unnecessarily reduce the number of cases. The strict quality criteria of the chosen radiographs should make gross measurement errors unlikely. Overall nonparametric tests do not rely on the removal of outlier, so that no further reduction of cases was necessary. Furthermore, the recruitment of a sufficient number of cases was highly challenging due to the narrow inclusion corridor and many elder patients were not available for a follow-up survey. Despite these obstacles, the basic scientific idea of reproducibly accurate data collection was to be maintained. For this very reason, strict inclusion criteria were essential, especially with regard to the quality of the radiographs. Afterwards, a suitable case number was achieved to present significant results.

### Language

Fluent English-speaking authors, some of whom are native speakers, wrote this text. This process was also supported by DeepL Translator [www.DeepL.com/Translator (free version)].

## Results

Medialization of the COR has a significantly negative effect on the need for care (Barthel Index) at patient discharge (Table 2, Spearman correlation RC-ML 0.357, *p* = 0.013; Spearman correlation RC-MLI 0.353, *p* = 0.014). This effect on patient mobility can still be demonstrated in a very significant manner using the PMS at 6 and 12 months (Table 2, Spearman correlation at 6 months RC-ML 0.471, *p* = 0.009; Spearman correlation at 12 months RC-ML 0.472, *p* = 0.008). Mann–Whitney *U* tests showed that the groups with medialized COR performed significantly worse than the lateralized groups. This was seen via the Barthel Index at discharge and at 6 months after surgery and the PMS at 6 and 12 months (Table 3). The accurately reconstructed CORs showed no significant difference from the lateralized rotation centers in terms of need of care and mobility (Table 4).

**Table 2:** Spearman correlations of COR reconstruction with the Barthel Index, PMS, and NRS

		RC-ML (mm)	RC-MLI	RC-SI (mm)	CPS (mm)	ALL (mm)
Barthel Index upon discharge	Cor	0.357	0.353	0.008	0.302	0.059
	*p*-Value	**0.013**	**0.014**	0.947	**0.041**	0.632
	*N*	48	48	64	46	68
Barthel Index after 6 months	Cor	0.366	0.368	−0.066	0.223	−0.167
	*p*-Value	0.06	0.059	0.706	0.284	0.322
	*N*	27	27	35	25	37
Barthel Index after 12 months	Cor	0.315	0.315	0.131	0.339	−0.173
	*p*-Value	0.11	0.11	0.452	0.097	0.305
	*N*	27	27	35	25	37
PMS after 6 months	Cor	0.471	0.469	0.169	0.477	0.032
	*p*-Value	**0.009**	**0.009**	0.309	**0.01**	0.847
	*N*	30	40	38	30	40
PMS after 12 months	Cor	0.472	0.462	0.415	0.67	0.106
	*p*-Value	**0.008**	**0.01**	**0.01**	** < 0.001**	0.513
	*N*	30	30	38	28	40
NRS after 6 months	Cor	−0.212	−0.207	0.231	0.135	0.09
	*p*-Value	0.245	0.256	0.164	0.477	0.582
	*N*	32	32	38	30	40
NRS after 12 months	Cor	−0.235	−0.225	0.17	0.03	0.042
	*p*-Value	0.188	0.208	0.299	0.872	0.795
	*N*	33	33	39	31	41

Significant *p*-values are highlighted using bold font

**Table 3:** Mann–Whitney *U* test between groups medialized (med) and lateralized (lat) CORs by more than 2 mm

	Groups	*N*	Mean rank	*p*-Value
Barthel Index upon discharge	Med	15	14.3	**0.029** (two-sided)
	Lat	22	22.2	**0.028** (one-sided significance)
	Total	37		
Barthel Index after 6 months	Med	10	8.75	**0.028** (two-sided)
	Lat	13	14.5	**0.042** (one-sided significance)
	Total	23		
Barthel Index after 12 months	Med	10	10.15	0.176 (two-sided)
	Lat	13	13.42	0.257 (one-sided significance)
	Total	23		
PMS after 6 months	Med	12	10.29	**0.036** (two-sided)
	Lat	14	16.25	**0.046** (one-sided significance)
	Total	26		
PMS after 12 months	Med	12	9.79	**0.012** (two-sided)
	Lat	14	16.68	**0.02** (one-sided significance)
	Total	26		
NRS after 6 months	Med	12	16.63	0.228 (two-sided)
	Lat	16	12.91	0.241 (one-sided significance)
	Total	28		
NRS after 12 months	Med	13	16.38	0.413 (two-sided)
	Lat	16	13.88	0.449 (one-sided significance)
	Total	29		

Significant results are highlighted using bold font

**Table 4:** Mann–Whitney *U* test between groups lateralized (lat) by more than 2 mm and with exact (exact) COR-reconstruction within ± 2 mm

	Groups	*N*	Mean rank	*p*-Value
Barthel Index upon discharge	Lat	22	21.75	0.141 (two-sided)
	Exact	16	16.41	0.145 (one-sided significance)
	Total	38		
Barthel Index after 6 months	Lat	13	14.38	0.253 (two-sided)
	Exact	12	11.5	0.347 (one-sided significance)
	Total	25		
Barthel Index after 12 months	Lat	13	14.08	0.355 (two-sided)
	Exact	12	11.83	0.47 (one-sided significance)
	Total	25		
PMS after 6 months	Lat	14	13.89	0.745 (two-sided)
	Exact	12	13.04	0.781 (one-sided significance)
	Total	26		
PMS after 12 months	Lat	14	14.75	0.269 (two-sided)
	Exact	12	12.04	0.374 (one-sided significance)
	Total	26		
NRS after 6 months	Lat	16	15.16	0.347 (two-sided)
	Exact	11	12.32	0.368 (one-sided significance)
	Total	27		
NRS after 12 months	Lat	16	14.91	0.449 (two-sided)
	Exact	11	12.68	0.481 (one-sided significance)
	Total	27		

Significant results are highlighted using bold font

The superior COR placement group showed significantly reduced mobility at 12 months in contrast to the inferior COR placement group (Table 5, *p* = 0.008), and the group of accurately reconstructed rotation centers showed significantly less pain than the inferior COR placement group (Table 6, *p* = 0.007 after 6 months, *p* = 0.026 after 12 months). With regards to mobility, these groups differed only in the two-sided significance test, but not when the one-sided significance test was used due to a limited number of cases. It narrowly failed our established significance level (Table 6). A possible statistically interfering influence of the leg length cannot be attested, as leg length showed no significant influence on mobility and need of care in this study (Table 2).

**Table 5:** Mann–Whitney *U* test between groups with superior COR placement (sup) and inferior COR placement (inf) COR by more than 2 mm

	Groups	*N*	Mean rank	*p*-Value
Barthel Index upon discharge	Sup	27	18.72	0.442 (two-sided)
	Inf	8	15.56	0.451 (one-sided significance)
	Total	35		
Barthel Index after 6 months	Sup	15	12.23	0.797 (two-sided)
	Inf	9	12.94	0.815 (one-sided significance)
	Total	24		
Barthel Index after 12 months	Sup	15	11.9	0.543 (two-sided)
	Inf	9	13.5	0.599 (one-sided significance)
	Total	24		
PMS after 6 months	Sup	17	13.06	0.396 (two-sided)
	Inf	10	15.6	0.443 (one-sided significance)
	Total	27		
PMS after 12 months	Sup	17	10.97	**0.004** (two-sided)
	Inf	10	19.15	**0.008** (one-sided significance)
	Total	27		
NRS after 6 months	Sup	18	13.03	0.105 (two-sided)
	Inf	11	18.23	0.112 (one-sided significance)
	Total	29		
NRS after 12 months	Sup	18	13.72	0.287 (two-sided)
	Inf	11	17.09	0.317 (one-sided significance)
	Total	29		

Significant results are highlighted using bold font

**Table 6:** Mann–Whitney *U* test between groups with inferior COR placement (inf) by more than 2 mm and exact COR-reconstruction (exact) within ± 2 mm

	Groups	*N*	Mean rank	*p*-Value
Barthel Index upon discharge	Inf	8	17.75	0.391 (two-sided)
	Exact	33	21.79	0.409 (one-sided significance)
	Total	41		
Barthel Index after 6 months	Inf	9	12.83	0.845 (two-sided)
	Exact	15	12.3	0.861 (one-sided significance)
	Total	24		
Barthel Index after 12 months	Inf	9	13.22	0.644 (two-sided)
	Exact	15	12.07	0.726 (one-sided significance)
	Total	24		
PMS after 6 months	Inf	10	12.75	0.88 (two-sided)
	Exact	15	13.17	0.892 (one-sided significance)
	Total	25		
PMS after 12 months	Inf	10	16.1	**0.038** (two-sided)
	Exact	15	10.93	0.091 (one-sided significance)
	Total	25		
NRS after 6 months	Inf	11	16.59	**0.008** (two-sided)
	Exact	13	9.04	**0.007** (one-sided significance)
	Total	24		
NRS after 12 months	Inf	11	16	**0.02** (two-sided)
	Exact	13	9.54	**0.026** (one-sided significance)
	Total	24		

The CPS showed a significant influence on the Barthel Index at discharge (Table 7: Spearman correlation 0.302; *p* = 0.041), as well as a very significant influence on the PMS at 6 months and a highly significant influence on the PMS at 12 months (Table 7: Spearman correlation at 6 months 0.477; *p* = 0.01; Spearman correlation at 12 months 0.67; *p* =  < 0.001). In the Mann–Whitney *U* test performed, increased CPS, and thus increased superomedial milling depth, showed significantly worse mobility, as measured by PMS, 12 months postoperatively (Table 7, two-sided *p* = 0.003).

**Table 7:** Mann–Whitney *U* test between groups with positive (pos) and negative (neg) CPS

	Groups	*N*	Mean rank	*p*-Value
Barthel Index upon discharge	Neg	33	23.85	0.095 (two-sided)
	Pos	19	31.11	
	Total	52		
Barthel Index after 6 months	Neg	21	16.69	0.316 (two-sided)
	Pos	14	19.96	0.359 (one-sided significance)
	Total	35		
Barthel Index after 12 months	Neg	21	16.69	0.283 (two-sided)
	Pos	14	19.96	0.359 (one-sided significance)
	Total	35		
PMS after 6 months	Neg	23	17.22	0.093 (two-sided)
	Pos	15	23	0.121 (one-sided significance)
	Total	38		
PMS after 12 months	Neg	23	15.61	**0.003** (two-sided)
	Pos	15	25.47	**0.007** (one-sided significance)
	Total	38		
NRS after 6 months	Neg	22	17.36	0.093 (two-sided)
	Pos	17	23.41	0.104 (one-sided significance)
	Total	39		
NRS after 12 months	Neg	23	18.89	0.29 (two-sided)
	Pos	17	22.68	0.315 (one-sided significance)
	Total	40		

Significant results are highlighted using bold font

## Discussion

COR medialization, superior COR displacement, and especially superomedialization leads to reduced recovery of mobility. Likewise, inferior COR displacement leads to increased pain. The results of the follow-up survey indicate that these are long-term effects. It is therefore recommended that vertical displacement in both directions and medialization of the center of rotation must always be avoided. A reasonable cause could be a resulting abductor weakness [14, 15]. The negative clinical effects of medial as well as superior displacements of the COR, which we have proven, also seemed to be biomechanically decisive in experimental tests using 3D models of dysplastic hips, whereas anterior–posterior displacements of the COR are not functionally relevant [14]. It has also been experimentally demonstrated that superolateralization of the COR increases hip joint forces compared with superior COR placement alone [20]. In the past, another approach to improve the long-term functional outcome was to reduce hip loading forces. From this point of view, an inferior placement and medialization of the COR seemed to be advantageous [16]. More recently, Asayama et al. also recommended inferomedialization of the center of rotation to improve abductor forces [15]. However, these recommendations should be viewed critically according to our clinical results. Such a COR position can only be achieved by a strongly medialized milling direction and an increased cup size. Our results also suggest that although improved function is achieved by inferior COR displacement, increased pain would be expected (Table 6). We therefore recommend an exact vertical restoration of the COR while avoiding functionally unfavorable medialization at all costs (Table 2). In addition to the functional effects observed in this study, inaccurate reconstruction of the COR seems to be a decisive predictor of the tendency to dislocation [21]. Furthermore, it is hypothesized that in addition to functional effects, wear and tear of the prosthesis will increase with inaccurate COR reconstruction [22, 23]. However, it also shows that the cup inclination seems to have the significant influence here and not COR reconstruction [24], which invalidates the recommendation for inferior displacement and medialization to reduce the above-mentioned loading forces. Rather, the combination of COR reconstruction in combination with unfavorable acetabular inclinations requires detailed consideration.

Increased milling depth tends to result in an increased medial and superior displacement of the COR (Table 1). This cannot be compensated by a higher femoral offset [25]. This is consistent with our philosophy that COR reconstruction represents the biomechanically sensitive joint plane and offset reconstruction rather influences soft tissue balancing and the lever arms of the muscles effecting the joint. The introduction of the CPS in the context of this study therefore seems logical and led to the detection of more significant correlations with regard to mobility than in the analyses with monodirectional measurements. Overall, however, it can be concluded that a superomedialization of the COR tends to be achieved in the arthroplastic treatment of medial femoral neck fractures (Table 1). We anticipate that knowledge of the results of our study will further improve the quality of care at our center. Therefore, from our point of view, a similar evaluation at other trauma centers and arthroplasty centers is recommended in order to gain knowledge for further optimization via similar quality assuring measures and statistical analysis. The results are also relevant considering current studies suggesting that immediate revision is safe for correcting radiological abnormalities [26].

An important point to mention is that the acetabular implants we primarily use do not offer a lateralized inlay on the European market. Therefore, the clinical results determined by us should also be urgently discussed among manufacturers, as this surgical option presumably improves the quality of life of some patients.

This study has some limitations. Due to a strict radiological quality-oriented selection of patients with evaluable X-ray images, in particular under strict avoidance of significant rotation errors, a smaller number of cases than planned could be achieved for the analyses. However, due to the precise measurement of the X-ray images, significant correlations could still be made visible. Furthermore, the limited accessibility by telephone of the elderly patients had a negative effect on the number of cases. Due to the limited follow-up potential of this patient group, we therefore recommend a strict selection of radiological images also for future studies, even if a lower number of cases must be accepted, as accurate measurements are the only way to make clinically significant results visible even with smaller numbers of cases. It also reduces the statistical influence of confounding effects.

For the evaluation of the follow-up survey, only patients with full scores on the PMS (9/9) or Barthel Index (100/100) were included to minimize the influence of preoperative limitations on the static analyses. Although this measure also contributed to smaller case numbers, it made statically significant correlations visible when these confounding influences were avoided.

## Conclusions

We recommend an exact vertical restoration of the COR while avoiding functionally unfavorable horizontal medial COR-displacement at all costs. COR medialization, superior COR displacement, and especially the combination of both (superomedialization) leads to reduced mobility while inferior displacement leads to increased pain. Therefore, vertical COR reconstruction needs to be exact, while medialization of COR must be strictly avoided. COR lateralization seems to be acceptable. However, superomedialization of COR tends to be achieved in THA of medial femoral neck fractures. An increased milling depth tends to result in increased medial and superior displacement of the COR. The introduction of the CPS in the context of this study therefore seems logical and led to the detection of more significant correlations with mobility than monodirectional measurements. Lateralized inlays should be a part of every THA implant portfolio. Sadly, this option is not yet widely available by some manufacturers. Our clinical results call for further discussion among and with manufacturers, as surgeons and patients rely on this option.

## Data Availability

The datasets used and/or analysed during the current study are available from the corresponding author on reasonable request.

## Abbreviations

COR: Center of rotation
PMS: Parker mobility score
THA: Total hip arthroplasty
HHA: Hemi hip arthroplasty
RC-ML: Horizontal center-of-rotation position
RC-MLI: Indexed horizontal center-of-rotation position
RC-SI: Vertical center-of-rotation position
ALL: Anatomical leg length
NRS: Numeric rating scale
CPS: Center of rotation position score
